# Complete Mitochondrial Genome of *Acartiella sinensis* Shen & Lee, 1963 (Copepoda, Calanoida) and Its Phylogeny

**DOI:** 10.3390/ani16152319

**Published:** 2026-07-28

**Authors:** Quehui Tang, Lianggen Wang, Yafang Li, Jiajia Ning, Shuangshuang Liu, Delian Huang, Jialiang Zhu, Xiaoqing Wu, Feiyan Du

**Affiliations:** 1 South China Sea Fisheries Research Institute, Chinese Academy of Fishery Sciences, Guangzhou 510300, China; 2 The Key Laboratory of Water Environment Simulation and Pollution Control, Ministry of Ecology and Environment of the People’s Republic of China, Guangzhou 510535, China; 3 South China Institute of Environmental Science, Ministry of Ecology and Environment of the People’s Republic of China, Guangzhou 510535, China

**Keywords:** Calanoida, Acartiidae, *Acartiella sinensis*, mitochondrial genome, gene rearrangement, phylogeny

## Abstract

Acartiidae are often among the most dominant and ecologically critical copepod groups in estuarine pelagic food webs. Here, we sequenced and characterized the complete mitochondrial genome of *Acartiella sinensis*, representing the first mitogenome for the family Acartiidae. The circular genome is 14,368 bp in length and exhibits a strong A+T bias (79.7%) and a compact organization, with all genes encoded on the heavy strand. Codon usage exhibits a preference for A/U-ending codons, while tRNA secondary structure analysis reveals two serine tRNAs lacking DHU arms and several G–U mismatches. Comparative analysis across 29 copepod mitogenomes indicates a distinctive gene arrangement in *A. sinensis*, particularly a reversal of *Cytb* and *NAD3* with local tRNA rearrangements. Phylogenetic analyses based on concatenated sequences of 13 protein-coding genes recover *A. sinensis* as sister to the clade comprising *Labidocera rotunda* (Pontellidae) and *Eurytemora affinis* (Temoridae), supporting close evolutionary relationships among these calanoid lineages. Our study provides fundamental genomic information for *A. sinensis* and contributes to the understanding of mitochondrial genome evolution and phylogenetic relationships within Calanoida.

## Simple Summary

Acartiidae are often among the most dominant and ecologically critical copepod groups in estuarine pelagic food webs. Here, we characterized the complete mitochondrial genome of *Acartiella sinensis*, representing the first mitogenome for the family Acartiidae. The complete mitochondrial genome of *A. sinensis* is 14,368 bp in length with 79.7% A+T. All genes are encoded on the heavy strand with A/T-biased nucleotide composition and atypical tRNA structures. Compared to 29 copepod mitogenomes, it shows a unique gene arrangement, with the *Cytb* and *NAD3* genes exchanged in position and neighboring tRNA genes locally rearranged. Phylogenetic analyses place *A. sinensis* as a sister to *Labidocera rotunda* + *Eurytemora affinis*, clarifying calanoid relationships and providing key genomic data for estuarine copepod evolution.

## 1. Introduction

Complete mitochondrial genomes provide rich and high-resolution genetic data for phylogenetic and phylogeographic analyses [1,2,3]. In recent years, such data have been extensively applied to phylogenetic and comparative genomic studies of aquatic crustaceans, offering deeper insights into their taxonomy and evolutionary history [4,5,6]. Copepods are among the most diverse and abundant microcrustacean groups and play a crucial role in marine pelagic food webs [7,8]. Given this ecological importance, complete mitochondrial genomes have been sequenced for 28 copepod species, spanning the families Calanidae, Diaptomidae, Pontellidae, Pseudodiaptomidae, Temoridae, Cyclopettidae, Ergasilidae, Lernaeidae, Harpacticidae, Miraciidae, Paramesochridae, Lernanthropidae, Pandaridae, and Pennellidae. However, no mitochondrial genome has yet been reported for the family Acartiidae, despite its broad distribution in estuaries and coastal waters [9,10,11].

Many species within the family Acartiidae exhibit strong adaptability to a wide range of salinity and nutrient conditions, allowing them to dominate copepod communities in estuaries characterized by substantial environmental fluctuations [9,12,13,14]. To date, studies on Acartiidae have focused on morphological classification [15,16], geographic distribution [17,18], and community ecology [9,11]. Molecular investigations, however, remain scarce and have relied exclusively on a limited set of genetic markers, such as COI and 18S rRNA [19,20]. The limited information provided by these short sequences constrains robust inference of phylogenetic relationships within the family.

In this study, we focused on *Acartiella sinensis* (Shen & Lee, 1963), a species within the family Acartiidae. Since its original description, this species has undergone multiple morphological redescriptions and taxonomic revisions [21,22]. *A. sinensis* is native to the southeastern coast of China and parts of Southeast Asia [22,23], and has been introduced to the west coast of the United States via human-mediated dispersal [24,25]. In light of its dominance in estuarine copepod communities, *A. sinensis* is of considerable ecological interest and can serve as a suitable model organism for investigating copepod adaptation to estuarine environments [23,26,27,28].

Here, we report the complete mitochondrial genome of *A. sinensis* as the first representative of the family Acartiidae. We perform comprehensive analyses of its genome structure, base composition, protein-coding gene characteristics, codon usage bias, tRNA secondary structures, and gene arrangement. Using published mitochondrial genome data from other copepods, we reconstruct phylogenetic relationships based on concatenated sequences of 13 protein-coding genes, employing both maximum likelihood (ML) and Bayesian inference (BI). This study fills the gap in mitochondrial genomic data for Acartiidae and clarifies the phylogenetic position of *A. sinensis* within Copepoda.

## 2. Materials and Methods

### 2.1. Sample Collection, Identification, and Sequencing

**Figure 1:**
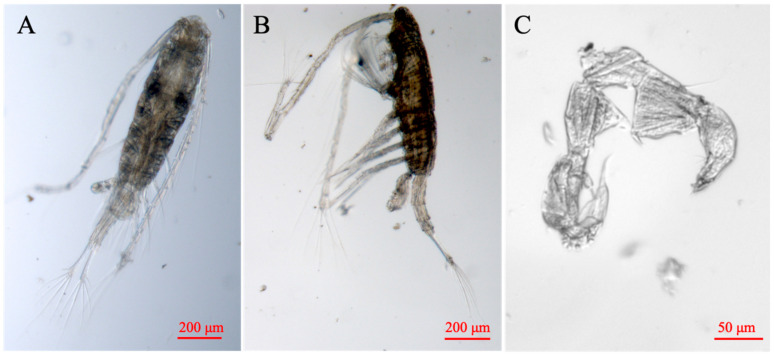
*A. sinensis* from the Pearl River Estuary, China. (**A**,**B**) Male individuals, (**C**) Fifth thoracic leg.

We collected the A. sinensis specimens in the Pearl River Estuary (22.88° N, 113.52° E) during November 2023. After collection, all specimens were immediately fixed in 95% ethanol and stored at −20 °C until further processing. Species identification was based on the morphological characteristics following Shen et al. [21], and representative photographs of the key taxonomic features are shown in Figure 1. A total of 50 intact male individuals were selected and preserved in absolute ethanol for genomic DNA extraction. Genomic DNA was extracted using the Rapid Genomic DNA Extraction Kit (Takara Bio Inc., Beijing, China) according to the manufacturer’s instructions. The quality of the extracted DNA was evaluated by 1% agarose gel electrophoresis. Qualified DNA samples were submitted to Nanjing Personalbio Technology (Nanjing, China) for library preparation and high-throughput sequencing. Paired-end sequencing with a read length of 150 bp was performed on the Illumina NovaSeq 6000 platform (Illumina, San Diego, CA, USA).

### 2.2. Mitochondrial Genome Assembly and Annotation

Raw reads were processed using fastp v0.23.4 [29] and Trimmomatic v0.39 [30] with default parameters for adapter removal, quality trimming, and length filtering, yielding over 10 GB of high-quality clean reads. We performed de novo assembly of the mitochondrial genome using both GetOrganelle [31] and NOVOPlasty [32], with the COI gene sequence of *A. sinensis* (GenBank accession no. KF977238.1) used as the seed sequence. In GetOrganelle, we conducted a de Bruijn graph-based assembly with k-mers of 21, 45, 65, 85, and 105 and a maximum of 20 iterations. In NOVOPlasty, we used a seed-extension-circularization strategy with a k-mer size of 33. Both assembly methods produced consistent circular mitochondrial genome sequences.

We performed gene annotation using MITOS2 through the Galaxy Europe platform (usegalaxy.eu, accessed on 19 May 2026) to predict the locations of protein-coding genes (PCGs), transfer RNAs (tRNAs), and ribosomal RNAs (rRNAs) [33]. The preliminary locations of PCGs were predicted by identifying open reading frames (ORFs) using the invertebrate mitochondrial genetic code, and the start and stop codons were verified through multiple sequence alignment with homologous PCGs from other copepod mitogenomes. The exact boundaries of the large and small ribosomal RNA genes (rRNAL and rRNAS) were defined through alignment with homologous sequences. We used MITOS to predict the secondary structures of all 22 tRNA genes for subsequent functional analysis.

### 2.3. Base Composition, Codon Usage, and Skew Analysis

We analyzed the complete mitochondrial genome of *A. sinensis* using PhyloSuite v1.2.3 [34]. We calculated nucleotide composition, relative synonymous codon usage (RSCU), and amino acid usage for all protein-coding genes. Strand compositional asymmetry was assessed by AT-skew and GC-skew values using the integrated functions of PhyloSuite.

### 2.4. Phylogenetic Analysis

Based on the nucleotide sequences of 13 protein-coding genes (PCGs), we conducted phylogenetic analyses to determine the phylogenetic position of *A. sinensis* within Copepoda. A total of 28 additional complete mitochondrial genomes of Copepoda were retrieved from GenBank (Table S1). The selected taxa included 7 Calanoida species, 10 Cyclopoida species, 7 Harpacticoida species, and 4 Siphonostomatoida species. Two *Daphnia* species (*D. galeata*, NC034297; *D. pulex*, NC000844) were used as outgroups.

We extracted nucleotide sequences of the 13 PCGs using PhyloSuite and performed multiple sequence alignment using MAFFT [35]. The resulting alignments were then refined using MACSE to correct for frameshifts and alignment errors caused by insertions/deletions [36]. We removed non-conserved regions and low-confidence sites using Gblocks [37]. The filtered gene sequences were concatenated into a final data matrix using the Concatenate Sequence function in PhyloSuite.

Maximum likelihood (ML) analyses were performed with IQ-TREE 3 [38]. The best-fit nucleotide substitution model for each partition was selected using ModelFinder, based on the Bayesian Information Criterion (BIC) [39], and partitioning was optimized using the MFP+MERGE strategy. Node support was assessed with 1000 SH-like approximate likelihood ratio tests (SH-aLRT) and 5000 ultrafast bootstrap replicates (UFBoot).

Bayesian inference (BI) analyses were conducted with MrBayes 3.2.7 [40]. Using the 13 partitions determined by IQ-TREE, the GTR+Gamma (GTR+G) model was applied uniformly, allowing independent estimation of base frequencies, substitution rates, and Gamma distribution parameters for each partition. Two independent runs were conducted, each with 4 Markov chains, running for 5,000,000 generations, sampling every 1000 generations, and performing convergence diagnostics every 5000 generations. The first 25% of samples were discarded as burn-in, and the remaining samples were used to construct a 50% majority-rule consensus tree with posterior probabilities (PP) calculated for each node. Convergence was assessed using the average standard deviation of split frequencies (ASDSF), with ASDSF < 0.01 indicating satisfactory convergence.

## 3. Results

### 3.1. Mitochondrial Genome Structure

**Figure 2:**
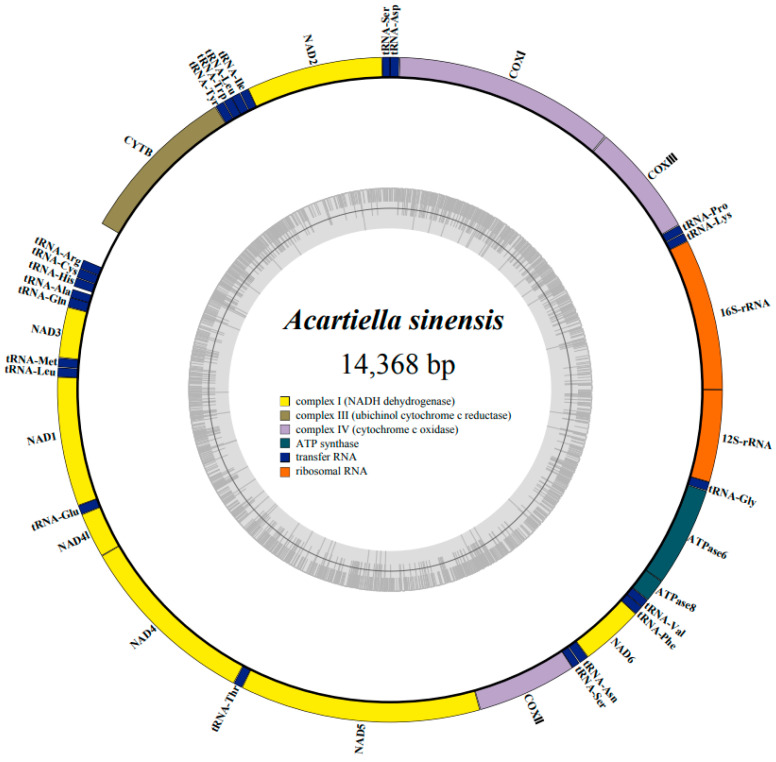
Mitochondrial genome maps of *A. sinensis*. The gray part of the inner circle represents GC content.

The complete mitochondrial genome of *A. sinensis* (GenBank accession no. PZ461130) is a circular molecule of 14,368 bp in length (Figure 2). It contained 37 mitochondrial genes including 13 PCGs, 2 rRNAs, and 22 tRNAs, together with a non-coding region (NCR) putatively corresponding to the control region (Figure 2; Table 1). All functional genes were located on the heavy (H-) strand. The mitogenome exhibited a highly compact gene arrangement, with 11 of the 36 adjacent gene pairs directly contiguous (i.e., no intergenic spacers or overlaps; Table 1). Most intergenic spacers were short, ranging from 1 to 4 bp, with the longest spacer of 19 bp located between *tRNA-His* and *tRNA-Ala*. Several adjacent gene pairs were directly contiguous, whereas gene overlaps were generally short, usually 1 or 2 bp in length. These overlaps mainly occurred between adjacent tRNA genes or between PCGs and tRNA genes (Table 1).

The overall nucleotide composition of the mitogenome was 35.3% A, 44.4% T, 11.7% G, and 8.6% C, with a high A+T content of 79.7% (Table 2). The AT-skew and GC-skew values were −0.113 and 0.154, respectively, indicating a nucleotide compositional bias toward T over A and G over C. The NCR was 277 bp and was located between *Cytb* and *tRNA-Arg*. Its nucleotide composition was 35.0% A, 36.5% T, 20.6% G, and 7.9% C, with an A+T content of 71.5% (Table 2).

### 3.2. Protein-Coding Genes and Codon Usage

The 13 PCGs comprised a total of 10,959 bp and encoded 3642 amino acids. Among them, *NAD5* was the longest PCG with a length of 1683 bp, whereas *ATPase8* was the shortest at 162 bp (Table 1 and Table 2). All PCGs started with an ATN codon (ATG, ATT, or ATA). For stop codons, eight genes including *COX3*, *NAD2*, and *NAD3* terminated with TAA, whereas *Cytb* and *NAD1* terminated with TAG. Incomplete stop codons were also detected, with *COX1* and *NAD6* ending with TA and *COX2* ending with a single T (Table 1).

**Figure 3:**
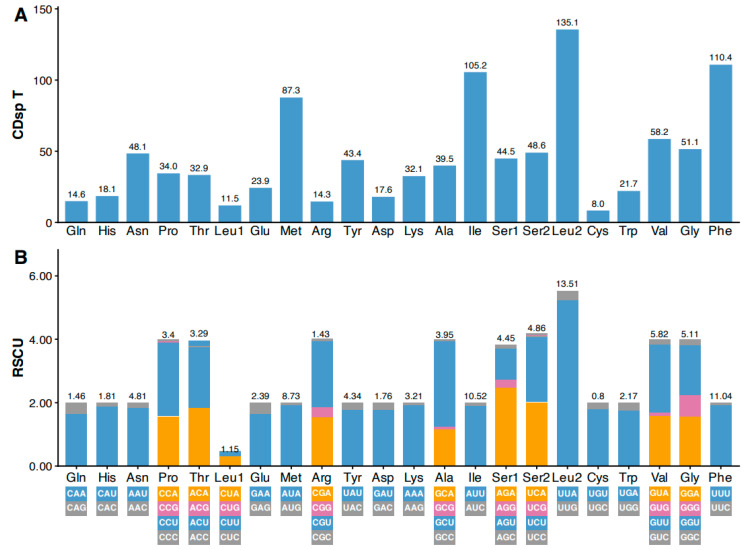
Amino acid usage and relative synonymous codon usage in the mitochondrial genome of *A. sinensis*. (**A**) Amino acid usage frequency in the 13 mitochondrial protein-coding genes. The x-axis represents amino acid types, and the y-axis represents the number of codons per thousand codons (CDspT). (**B**) Relative synonymous codon usage (RSCU) values of mitochondrial protein-coding genes. Different colors indicate codons with different third-position nucleotides.

Leucine (Leu) was the most frequently used amino acid in the mitochondrial PCGs of *A. sinensis*, accounting for 14.66% of total amino acids, followed by phenylalanine (Phe), at 11.04%. In contrast, cysteine (Cys) was the least frequently used amino acid, accounting for only 0.80% (Figure 3A). RSCU analysis revealed a pronounced codon usage bias. Most codons ending with adenine (A) or thymine (T) showed relatively high RSCU values, whereas most codons ending with guanine (G) or cytosine (C) exhibited lower values, consistent with the high A+T content of the mitogenome. Among all codons, UUA (leucine) exhibited the highest RSCU value (5.24), indicating that it was the most preferentially used codon (Figure 3B).

### 3.3. Ribosomal and Transfer RNA Genes

The two ribosomal RNA genes (*12S rRNA* and *16S rRNA*) comprised 1707 bp and exhibited nucleotide compositions of 44.2% A, 41.2% T, 8.8% G, and 5.8% C, resulting in a high A+T content (Table 2). The AT-skew and GC-skew values were 0.035 and 0.208, respectively. The *12S rRNA* (647 bp) and *16S rRNA* (1060 bp) were located between *tRNA-Gly* and *tRNA-Lys* and arranged in tandem (Table 1).

**Figure 4:**
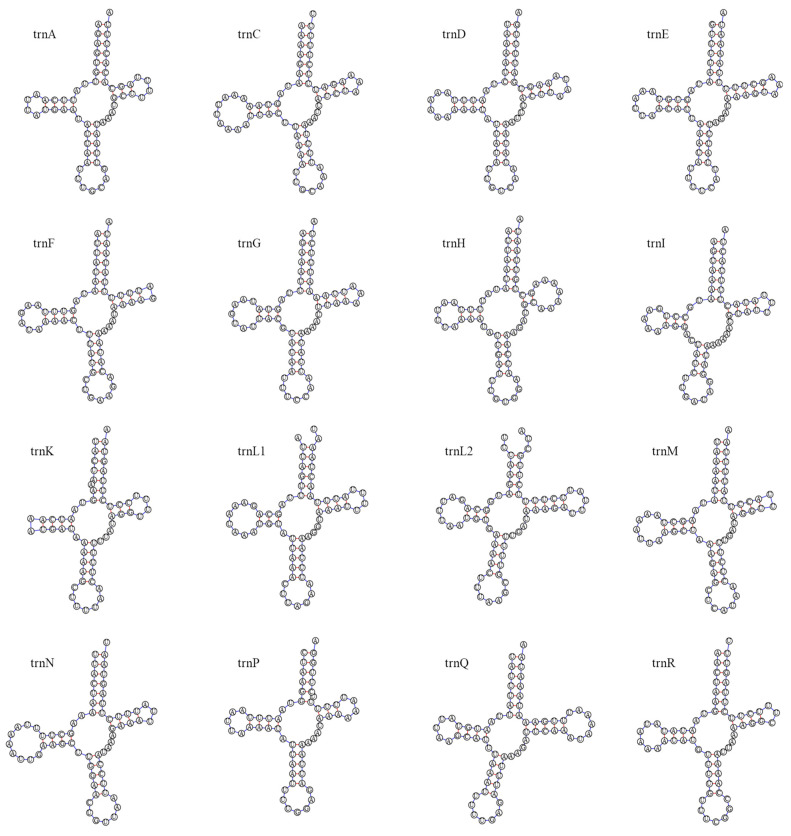
Predicted secondary structures of the 22 tRNAs in the mitochondrial genome of *A. sinensis*.

A total of 22 tRNA genes were identified, ranging from 56 bp (*tRNA-Ser*) to 69 bp (*tRNA-Gln*), with a total length of 1394 bp (Table 1 and Table 2). Most tRNAs (20 of 22) could be folded into typical cloverleaf secondary structures, whereas two serine tRNAs (*tRNA-Ser1* and *tRNA-Ser2*) lacked a dihydrouridine (DHU) arm, forming atypical structures (Figure 4). The anticodon stems were generally stable, consisting of 3–5 bp, and the acceptor stems were typically 7 bp, with slight variation in several tRNAs. A total of 16 G–U mismatches were detected across the tRNA genes (Figure 4). These mismatches were mainly distributed in the acceptor, DHU, and TΨC arms, whereas no mismatches were observed in the anticodon stems. Among all tRNAs, *tRNA-Glu* showed the highest number of mismatches (*n* = 3), followed by *tRNA-Leu2* and *tRNA-Ser1* (*n* = 2). Additionally, three tRNAs exhibited bulge structures. Single-nucleotide insertions were observed in *tRNA-Lys* and *tRNA-Pro* within the acceptor stem, and in *tRNA-Ser1* within the anticodon stem. Furthermore, the acceptor stems of *tRNA-Leu1* and *tRNA-Leu2* contained unpaired nucleotides at the termini.

### 3.4. Comparative Mitochondrial Gene Arrangement in Copepoda

**Figure 5:**
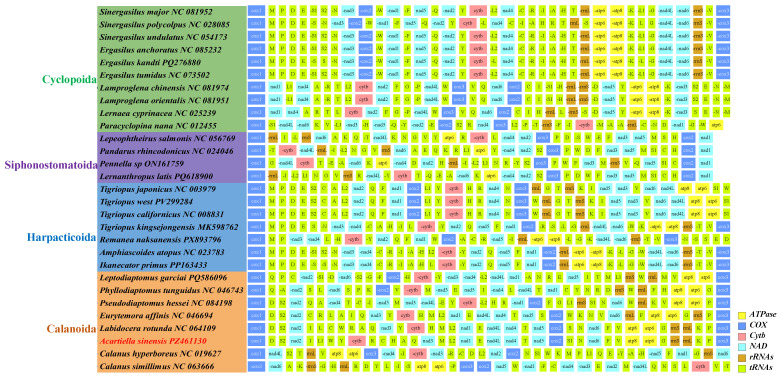
Comparative mitochondrial gene arrangement of *A. sinensis* and copepod species. Different colors indicate protein-coding genes, rRNA genes, and tRNA genes. Genes transcribed in the opposite direction are marked with a minus sign. Species are grouped by orders to highlight contrasting patterns of gene rearrangements within versus among orders.

We compared mitochondrial gene arrangements among 29 copepod species representing four orders (Calanoida, Harpacticoida, Cyclopoida, and Siphonostomatoida), with all genomes linearized from the *COX1* gene (Figure 5). Gene order was generally conserved within orders but differed substantially among orders. As the first reported mitogenome of Acartiidae, *A. sinensis* exhibited a gene arrangement distinct from those of other calanoid species. Compared with *Labidocera rotunda*, the order of most PCGs was conserved, although the relative positions of *Cytb* and *NAD3* were reversed with local rearrangements of neighboring tRNA genes. Compared with the species of Diaptomidae and Pseudodiaptomidae, more extensive rearrangements involving *Cytb*, *NAD1*, *NAD3*, *NAD4*, *NAD4L*, *NAD5*, and adjacent tRNA genes were observed in *A. sinensis*. The species of Siphonostomatoida displayed gene arrangements markedly different from those of Calanoida. While PCG order was relatively conserved within Siphonostomatoida, the positions of tRNA and rRNA genes, particularly *rrnL* and *rrnS*, varied substantially (Figure 5).

### 3.5. Phylogenetic Relationships Within Copepoda

**Figure 6:**
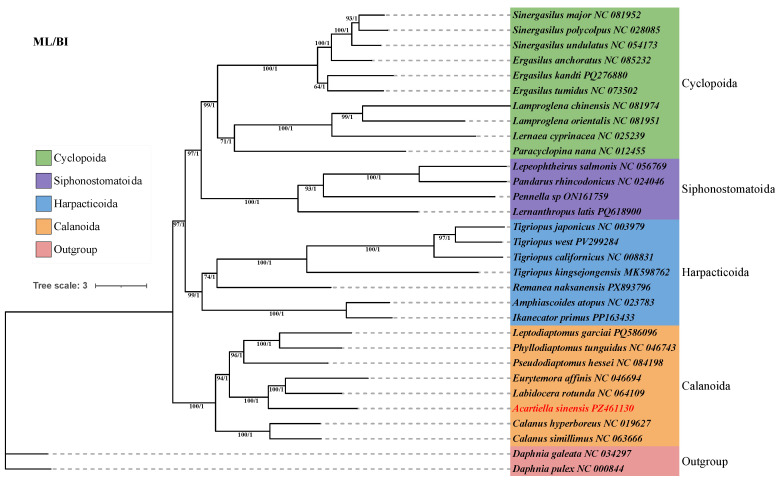
Phylogenetic relationships of 29 copepod species inferred from the nucleotide sequences of 13 mitochondrial protein-coding genes. Maximum likelihood (ML) and Bayesian inference (BI) methods produced identical topologies. Node support values (ultrafast bootstrap for ML and posterior probabilities for BI) are indicated at each node. Colors indicate different copepod orders: Calanoida (orange), Harpacticoida (blue), Cyclopoida (green), Siphonostomatoida (purple), and outgroups (Daphnia species, pink). *A. sinensis* is highlighted in red text.

Phylogenetic analyses used ML- and BI-generated identical topologies (Figure 6). All four copepod orders formed well-supported clades. Cyclopoida and Siphonostomatoida were sister groups and together formed a clade sister to Harpacticoida, whereas Calanoida occupied the basal position among the taxa analyzed. Within Calanoida, Calanidae represented the basal lineage, while Diaptomidae and Pseudodiaptomidae clustered as a derived clade. *A. sinensis* was recovered as sister to the clade comprising *L. rotunda* and *E. affinis*.

## 4. Discussion

Our study showed that *A. sinensis* exhibits conserved gene content but distinct structural and compositional features, including extreme A+T bias, strand asymmetry, and localized gene rearrangements. These characteristics, combined with phylogenetic evidence, advance our understanding of mitochondrial genome evolution in copepods and provide valuable genomic data for future studies on taxonomy, population genetics, and adaptive evolution within Acartiidae.

### 4.1. The Mitochondrial Genome of A. sinensis

The mitochondrial genome of *A. sinensis* contains a set of metazoan mitochondrial genes. This is in agreement with the conserved gene complement observed in most crustaceans [41,42,43]. The *A. sinensis* genome displays a highly compact organization characterized by extremely short intergenic spacers and occasional gene overlaps, which is typical of compact mitochondrial genomes in invertebrates [44]. Such compaction generally results from the selection pressure toward genome streamlining, leading to reduced non-coding regions and minimized genome size.

All mitochondrial genes of *A. sinensis* are encoded on the heavy strand. This feature has also been observed in some invertebrate lineages and has been reported in some arthropods such as millipedes [45]. The single-strand encoding pattern may represent a lineage-specific organizational strategy, potentially associated with replication and transcriptional asymmetry. In addition, the *A. sinensis* mitogenome exhibits a remarkably high A+T content (79.7%) and strong AT- and GC- skews, which are commonly observed in crustacean mitochondrial genomes [46,47]. Similar compositional biases have also been reported in other copepods such as *E. affinis* and *Calanus sinicus* [4,42]. These compositional biases may reflect long-term directional mutation pressure and strand-specific replication bias, which may have been influenced by the inversion events of replication origins in crustacean mitochondrial evolution [48].

The mitochondrial PCGs of *A. sinensis* initiate with ATN start codons, consistent with the mitochondrial translation system of invertebrates [47,49]. Most genes possess complete stop codons (TAA or TAG), whereas several exhibit incomplete termination codons. This is a widespread phenomenon in metazoan mitochondria. The incomplete stop codons are generally completed post-transcriptionally via polyadenylation, which restores functional termination signals while contributing to genome compaction [50]. Codon usage analysis reveals a strong bias toward A/U-ending codons, reflecting mutational pressure rather than translational selection. The leucine codon UUA is the most frequently used codon, indicating marked synonymous codon preference [46,47].

The ribosomal RNA genes of *A. sinensis* are comparable in length to those of other calanoid species, but their genomic arrangement differs markedly. In *A. sinensis*, the *12S rRNA* and 16S *rRNA* genes are tandemly located between *tRNA-Gly* and *tRNA-Lys*, whereas in other copepods such as *C. sinicus* and *Phyllodiaptomus tunguidus*, their positions are highly variable [4,5]. The rearrangements suggest the evolutionary plasticity of mitochondrial genome organization even among closely related taxa.

The 22 tRNA genes in *A. sinensis* mostly form canonical cloverleaf structures, although two *tRNA-Ser* genes lack the DHU arm. This structural reduction is considered a tolerated deviation in mitochondrial tRNAs [51,52,53]. Additionally, multiple G–U mismatches were detected across tRNAs, particularly in stem regions excluding anticodon loops. The non-canonical pairings contribute to the structural flexibility while maintaining functional stability [54,55].

Copepod mitochondrial genomes exhibit extensive gene rearrangements, although some conservation is still detectable at the order level [4,5,56]. Our comparative analysis across 29 copepod species confirms strong inter-order divergence but relative intra-order conservation. As the first mitochondrial genome reported for Acartiidae, *A. sinensis* provides important insight into calanoid genome evolution. Compared with *L. rotunda*, the overall gene order is largely conserved, except for a localized inversion in the *Cytb-NAD3* region accompanied by tRNA rearrangements. Such localized rearrangements may represent hotspots of genomic recombination or replication-related errors [57]. The rearrangements in comparisons with Diaptomidae and Pseudodiaptomidae further demonstrates the lineage divergence within Calanoida [58].

### 4.2. Phylogenetic Relationships Within Copepoda

Phylogenetic analyses based on concatenated mitochondrial PCGs yielded highly congruent topologies between ML and BI. All major copepod orders formed strongly supported clades [7,59]. Our results recover Calanoida as a basal lineage within Copepoda and confirm the split between Calanoida and Podoplea [4,60].

Within Podoplea, our results support the topology (Calanoida + (Harpacticoida + (Cyclopoida + Siphonostomatoida))), which agree with recent mitogenome-based phylogenies [60,61]. This topology differs from earlier morphological studies and analyses based on one or few rRNA genes, which often recovered Harpacticoida and Siphonostomatoida as sister groups [4,62]. Recent phylogenomic analyses based on hundreds or thousands of nuclear genes also support the monophyly of Podoplea and place Cyclopoida and Harpacticoida as sister groups, suggesting that higher-level copepod relationships are sensitive to molecular marker and dataset size [63]. Together with simulation and empirical studies on copepod mitogenomes, these findings underscore that sparse taxon sampling and short genetic fragments can lead to unstable interordinal relationships, whereas multilocus datasets with extensive sampling generally provide more robust phylogenetic signals [60].

Within Calanoida, our phylogenetic tree recovered Calanidae as a basal lineage and placed Diaptomidae and Pseudodiaptomidae as a derived clade [5,64]. Comparative mitogenomic analyses indicate that Diaptomidae species have relatively conserved PCG arrangements and similar genomic features, revealing the close evolutionary relationships within this lineage [5,43].

The phylogenetic position of Diaptomidae was consistent with previous mitogenomic studies. Specifically, previous analyses based on concatenated mitochondrial protein-coding genes recovered a strongly supported clade comprising *L. rotunda* and *E. affinis*, which formed the sister group to Diaptomidae species [57]. This close relationship was also supported by the analyses of concatenated mitochondrial PCGs from multiple copepod mitogenomes [58].

## 5. Conclusions

This study reports the complete mitochondrial genome of *A. sinensis*, representing the first mitogenome for Acartiidae. The genome is compact, exhibits a strong AT bias, and comprises the standard set of 37 mitochondrial genes. Its protein-coding genes exhibit pronounced A/U-ending codon preferences, whereas the tRNA set includes two serine tRNAs lacking complete DHU arms and multiple non-canonical base pairs. Comparative analysis revealed extensive variation in mitochondrial gene order across Copepoda and identified a distinctive arrangement in *A. sinensis*, particularly around the *Cytb–NAD3* region. Phylogenetic analyses placed *A. sinensis* as sister to the clade comprising *L. rotunda* and *E. affinis*. These results provide new evidence for mitochondrial genome evolution and phylogenetic relationships within Calanoida and establish a foundation for future taxonomic, population genetic, and adaptive evolution studies of Acartiidae.

## Figures and Tables

**Table 1:** Features of the mitochondrial genome of *A. sinensis*.

Feature	Position		Length	Amino Acid	Codons		Anticodon	Intergenic Region	Strand
	From	To			Start	Stop			
*16S rRNA*	1	1060	1060						H
*tRNA-Lys*	1061	1120	60				TTT	0	H
*tRNA-Pro*	1123	1183	61				TGG	2	H
*COX3*	1186	1977	792	263	ATG	TAA		2	H
*COX1*	1982	3525	1544	514	ATT	TA(A)		4	H
*tRNA-Asp*	3528	3592	65				GTC	2	H
*tRNA-Ser*	3591	3646	56				TGA	−2	H
*NAD2*	3647	4600	954	317	ATA	TAA		0	H
*tRNA-Ile*	4600	4661	62				GAT	−1	H
*tRNA-Leu*	4664	4728	65				TAG	2	H
*tRNA-Trp*	4727	4789	63				TCA	−2	H
*tRNA-Tyr*	4791	4854	64				GTA	1	H
*Cytb*	4856	5989	1134	377	ATG	TAG		1	H
NCR	5990	6266	277					0	H
*tRNA-Arg*	6267	6330	64				TCG	0	H
*tRNA-Cys*	6331	6397	67				GCA	0	H
*tRNA-His*	6399	6462	64				GTG	1	H
*tRNA-Ala*	6482	6542	61				TGC	19	H
*tRNA-Gln*	6541	6609	69				TTG	−2	H
*NAD3*	6610	6963	354	117	ATT	TAA		0	H
*tRNA-Met*	6965	7028	64				CAT	1	H
*tRNA-Leu*	7031	7097	67				TAA	2	H
*NAD1*	7097	7999	903	300	ATA	TAG		−1	H
*tRNA-Glu*	7999	8064	66				TTC	−1	H
*NAD4L*	8065	8379	315	104	ATA	TAA		0	H
*NAD4*	8380	9657	1278	425	ATA	TAA		0	H
*tRNA-Thr*	9659	9720	62				TGT	1	H
*NAD5*	9721	11,403	1683	560	ATA	TAA		0	H
*COX2*	11,405	12,104	700	233	ATT	T(AA)		1	H
*tRNA-Ser*	12,105	12,162	58				TCT	0	H
*tRNA-Asn*	12,166	12,232	67				GTT	3	H
*NAD6*	12,233	12,678	446	148	ATA	TA(A)		0	H
*tRNA-Phe*	12,678	12,740	63				GAA	−1	H
*tRNA-Val*	12,740	12,802	63				TAC	−1	H
*ATPase8*	12,803	12,964	162	53	ATT	TAA		0	H
*ATPase6*	12,964	13,659	696	231	ATG	TAA		−1	H
*tRNA-Gly*	13,661	13,723	63				TCC	1	H
*12S rRNA*	13,722	14,368	647					−2	H

**Table 2:** Nucleotide composition and skewness of different regions and genes in the mitochondrial genome of *A. sinensis*.

Gene	A%	T(U)%	G%	C%	(A+T)%	AT-skew	GC-skew	Length (bp)
Full genome	35.3	44.4	11.7	8.6	79.7	−0.113	0.154	14,368
PCGs	33.1	45.5	12.2	9.3	78.6	−0.158	0.137	10,959
tRNAs	42	40.5	10.3	7.2	82.5	0.017	0.180	1394
rRNAs	44.2	41.2	8.8	5.8	85.4	0.035	0.208	1707
*ATPase6*	33.8	43.8	12.1	10.3	77.6	−0.130	0.077	696
*ATPase8*	42	48.8	3.7	5.6	90.8	−0.075	−0.200	162
*COX1*	29.5	41.8	17	11.7	71.3	−0.173	0.183	1544
*COX2*	32.9	40.2	15.2	11.7	73.1	−0.099	0.132	700
*COX3*	30.4	42.2	15.8	11.6	72.6	−0.162	0.152	792
*Cytb*	31	43.3	14.1	11.6	74.3	−0.165	0.100	1134
*NAD1*	31.7	46.6	12.2	9.5	78.3	−0.191	0.122	903
*NAD2*	40.4	48.6	5.9	5.1	89	−0.093	0.067	954
*NAD3*	29.4	52.8	9.3	8.5	82.2	−0.285	0.048	354
*NAD4*	32.6	49.1	10.6	7.8	81.7	−0.202	0.149	1278
*NAD4L*	35.2	46.3	10.8	7.6	81.5	−0.136	0.172	315
*NAD5*	34.6	46.1	10.9	8.4	80.7	−0.142	0.130	1683
*NAD6*	35.7	51.3	9.2	3.8	87	−0.180	0.414	446
NCR	35	36.5	20.6	7.9	71.5	−0.020	0.443	277
*16S rRNA*	46.2	40.4	7.8	5.6	86.6	0.068	0.169	1060
*12S rRNA*	40.8	42.5	10.5	6.2	83.3	−0.020	0.259	647

## Data Availability

The datasets presented in this study can be found in online repositories. The names of the repository/repositories and accession number(s) can be found below: https://www.ncbi.nlm.nih.gov/genbank/PZ461130 (These data can be accessed after 31 December 2026).

## Supplementary Materials

The following supporting information can be downloaded at: https://www.mdpi.com/article/10.3390/ani16152319/s1, Table S1: List of species used to construct the phylogenetic tree.
