# Supplementary material for: Complete Mitochondrial Genome of Acartiella sinensis Shen & Lee, 1963 (Copepoda, Calanoida) and Its Phylogeny
Source: Animals (Basel). 2026 Jul 28;16(15):2319. doi: 10.3390/ani16152319 (PMC13463582; doi:10.3390/ani16152319)
Supplement: Supplementary file 1 [file animals-16-02319-s001.zip › animals-4418637-supplementary.pdf]

Table S1 List of species used to construct the phylogenetic tree.

Order	Family	Species	GenBank number
Calanoida	Acartiidae	*Acartiella sinensis*	PZ461130
Calanoida	Calanidae	*Calanus hyperboreus*	NC_019627
Calanoida	Calanidae	*Calanus simillimus*	NC_063666
Calanoida	Diaptomidae	*Leptodiaptomus garciai*	PQ586096
Calanoida	Diaptomidae	*Phyllodiaptomus tunguidus*	NC_046743
Calanoida	Pontellidae	*Labidocera rotunda*	NC_064109
Calanoida	Pseudodiaptomidae	*Pseudodiaptomus hessei*	NC_084198
Calanoida	Temoridae	*Eurytemora affinis*	NC_046694
Cyclopoida	Cyclopettidae	*Paracyclopina nana*	NC_012455
Cyclopoida	Ergasilidae	*Ergasilus anchoratus*	NC_085232
Cyclopoida	Ergasilidae	*Ergasilus kandti*	PQ276880
Cyclopoida	Ergasilidae	*Ergasilus tumidus*	NC_073502
Cyclopoida	Ergasilidae	*Sinergasilus major*	NC_081952
Cyclopoida	Ergasilidae	*Sinergasilus polycolpus*	NC_028085
Cyclopoida	Ergasilidae	*Sinergasilus undulatus*	NC_054173
Cyclopoida	Lernaeidae	*Lamproglena chinensis*	NC_081974
Cyclopoida	Lernaeidae	*Lamproglena orientalis*	NC_081951
Cyclopoida	Lernaeidae	*Lernaea cyprinacea*	NC_025239
Harpacticoida	Harpacticidae	*Tigriopus californicus*	NC_008831
Harpacticoida	Harpacticidae	*Tigriopus japonicus*	NC_003979
Harpacticoida	Harpacticidae	*Tigriopus kingsejongensis*	MK598762
Harpacticoida	Harpacticidae	*Tigriopus west*	PV299284
Harpacticoida	Miraciidae	*Amphiascoides atopus*	NC_023783
Harpacticoida	Miraciidae	*Ikanecator primus*	PP163433
Harpacticoida	Paramesochridae	*Remanea naksanensis*	PX893796
Siphonostomatoida	Caligidae	*Lepeophtheirus salmonis*	NC_056769
Siphonostomatoida	Lernanthropidae	*Lernanthropus latis*	PQ618900
Siphonostomatoida	Pandaridae	*Pandarus rhincodonicus*	NC_024046
Siphonostomatoida	Pennellidae	*Pennella* sp	ON161759
Anomopoda	Daphniidae	*Daphnia galeata*	NC_034297
Anomopoda	Daphniidae	*Daphnia pulex*	NC_000844
